# Empowering traditional birth attendants as agents of maternal and neonatal immunization uptake in Nigeria: a repeated measures design

**DOI:** 10.1186/s12889-021-10311-z

**Published:** 2021-02-04

**Authors:** Chinedu Anthony Iwu, Kenechi Uwakwe, Uche Oluoha, Chukwuma Duru, Ernest Nwaigbo

**Affiliations:** Department of Community Medicine, Imo State University, Teaching Hospital Umuna, Orlu, Nigeria

**Keywords:** Traditional birth attendants, Immunization uptake, Intervention, Nigeria

## Abstract

**Background:**

Adequate immunization coverage in rural communities remain a challenge in Nigeria. Traditional birth attendants (TBAs) form an integral part of the social, cultural and religious fabric in most rural communities in Nigeria. Despite their limitations in handling the complications of childbirth, TBAs are widely accepted and patronized, especially in rural areas. The objectives of the project were to empower TBAs and assess the use of a culturally adapted audio-visual workshop intervention to change their knowledge, attitude and willingness to promote immunization uptake.

**Methods:**

A repeated-measures design that used a convenience sampling technique to select 90 TBAs from the three geopolitical zones of Imo State, Nigeria. The TBAs were engaged through a culturally adapted audio-visual workshop. Data were collected before and immediately after intervention using a pretested questionnaire. Chi square test was done to determine any significant association with the zone of practice and paired sample t-test analysis to determine any significant pre and post intervention change. Level of significance was set at *p* ≤ ·05.

**Results:**

More than half of the TBAs had at most, a secondary level of education (54·4%). The average length of time they practiced as TBAs was 16 years with an average of ten birth deliveries per month. After the intervention, all the respondents (100%) reported a willingness to always promote immunization uptake and also, there was a statistically significant increase in Knowledge (*p* < ·000). Similarly, the level of knowledge in the post intervention period appeared to be significantly associated with the zone of practice (*p* = ·027).

**Conclusion:**

The workshop intervention empowered the TBAs irrespective of their zones of residence by successfully improving their knowledge, though at varying levels; and consequently, their willingness to always promote immunization uptake.

**Supplementary Information:**

The online version contains supplementary material available at 10.1186/s12889-021-10311-z.

## Background

Attaining optimal immunization coverage in the rural areas remain a challenge and this may be associated with uptake factors rooted in cultural, traditional and religious beliefs which may vary within different parts of a region or country. This situation of less than optimal coverage, observed especially in the rural communities may be compounded by the high proportion of home birth deliveries undertaken by traditional birth attendants (TBAs) as this reduces the contact of mothers with the health facilities where immunization sensitization and uptake usually takes place. Furthermore, contributing to this situation, may be the lack of awareness, incorrect information, misconception and vaccine safety concerns [1–3].

Globally, there is an observable gap in the proportion of births delivered in a health facility by skilled birth attendants between the high and low income countries. This gap may be due to the high community patronage of TBAs in low income countries, probably for reasons such as availability, affordability, accessibility and the TBA’s attitude of friendliness and care particularly observed [4, 5]. According to World Health Organization, TBAs are customary and autonomous of the health system with no formal training; and are community based providers of care during pregnancy, childbirth and the postpartum period [6]. In Nigeria, about 80% of the population live in rural conditions and according to Nigeria’s MDG end point report, women residing in rural areas were 77% more likely to give birth at home than in a health facility [7, 8]. This may also explain why the use of TBAs remains prevalent across the country despite the availability of skilled birth attendants who are trained and have relatively modern equipment [9].

It has been reported that, the TBAs when compared to the hospital based skilled attendants are inexpensive, culturally sensitive and compassionate when providing care and therefore, are trusted and respected within the communities [10]. As a consequence, special relationships develop between the community members and the TBAs. They become an integral part of the communities with wide spread acceptance across social, cultural and religious lines [11].

The training of TBAs in the conventional maternal health-care delivery systems, has shown to increase the utilization of health facility’s prenatal, antenatal and postnatal care services which invariably improves maternal and neonatal health [12]. Empowerment, a consequence of effective training is an intentional process, centered in local communities involving active participation, critical reflection, awareness, understanding and control over decisions [13].

In Nigeria’s Demographic Health Survey of 2018, it was reported that, rural women will give birth to about 1.4 children more than urban women during their reproductive years and are less likely to have received antenatal care from a skilled birth attendant [14]. This further emphasizes the potential roles that TBAs could play in the promotion of maternal and neonatal immunization when empowered through appropriate training. Therefore, the overall aim of this project is to empower TBAs by increasing knowledge, attitude and willingness to promote immunization through a culturally adapted audio-visual workshop in order to become agents of maternal and neonatal immunization uptake in rural communities.

## Methods

### Study area

The project was implemented in Imo State, which is situated in the South Eastern part of Nigeria within longitude 5°29′06“N and latitude 7°02’06”E occupying an area between the lower river Niger and the upper and middle Imo River [15]. It occupies an area of 5289 km^2^ with a total population of 3·93million (2·03 million males and 1·9million females) according to the 2006 census with an annual growth rate of 3·2% [16]. The State is delineated into 27 Local Government Areas and 305 registrations areas (communities) within three senatorial geopolitical zones; Owerri, Orlu and Okigwe with varying social development, cultural and traditional beliefs.

### Study population/sample size

The study population comprise traditional birth attendants practising within the three senatorial geopolitical zones of Imo State. The minimum sample size per zone for a two tailed paired sample T test analysis was calculated using G Power software version 3·1·9·4 where the estimated effect size of 0·5 based on a previous study [17], α value of 0·05 and a power of 80% was assumed. The minimum sample size calculated was 26 which was increased to 30 to accommodate incomplete or non-responses. A total study sample of 90 participants was enrolled (30 participants per senatorial geopolitical zone).

### Study design /sampling technique/selection criteria

The study was a repeated measures design where convenience sampling technique was used to select 90 TBAs. All TBAs practising within the State were invited and as they arrived, were enrolled according to their respective zones of practice until each zone attained a maximum of 30 participants. However, those that had received any formal medical training were excluded and replaced. The TBAs that arrived after enrolment had closed, were registered according to their zones but not enrolled. They formed the pool of TBAs from where replacements were randomly picked in cases of drop out or exclusion. TBAs were also randomly selected from the pool to participate in the pre-testing of the questionnaire.

### Workshop intervention/materials

The project commenced 7th January 2020 and was for a duration of 3 months. The workshop activities comprised content communication, health facility linkages and workshop evaluation. Workshop content was communicated through audio visual presentations comprising slide shows on the clinical effects of non-immunization and a drama production depicting the role of TBAs in the promotion of immunization uptake. The audio-visual content was developed by the project team and structured for the target audience by taking into consideration their level of literacy, language barriers, customs and traditions. Health facility linkages were achieved through health-linkage sensitization talks given by health facility immunization focal persons. The accessible health facilities that conduct immunization activities within the three geopolitical zones of the State were identified. Immunization focal persons from these facilities were invited and through health-linkage sensitization talks were engaged with the TBAs. The intention was to establish sustainable communication channels after the programme. The workshop evaluation involved the administration of a semi-structured questionnaire pre and post workshop. See questionnaire supplementary file. The questionnaire was developed by the project team and pretested among the pool of TBAs not enrolled to participate in the workshop. The content validity was established and a reliability coefficient (alpha) of 0·83 was calculated.

### Data collection/analysis

Data was collected using a pretested semi-structured interviewer-administered questionnaire. Research assistants were trained on the questionnaire administration and on the appropriate translations in the native language (Igbo). The questionnaire comprised 4 sections: one on socio-demographic characteristics; two on knowledge of maternal and neonatal immunizations; three on attitude towards maternal and neonatal immunizations and four on practices with respect to willingness to encourage clients on immunization uptake.

The level of knowledge was determined by scoring the questions that assessed knowledge. A correct answer was scored 2 and incorrect answer was scored 0. The aggregate knowledge scores for each respondent was assessed against a scale of ≤30 for poor, 31–38 for moderate and 39–46 for good knowledge of immunization. In assessing the level of attitude, an answer connoting a positive attitude was scored 2 and a negative attitude was scored 0 and a “no opinion” attitude was scored 1.The aggregate attitude scores for each respondent was assessed against a scale of ≤8 for poor, 9–10 for moderate and 11–12 for good attitude towards immunization.

Data was cleaned and validated manually then analysed using Software Package for Social Sciences (IBM-SPSS) version 22. Descriptive statistics (frequency tables and summary indices) were generated. Chi square test was done to determine any significant association with the zone of practice. Paired sample t-test analysis was done to determine any significant change in knowledge and attitude towards immunization. A level of significance was set at *p* ≤ ·05 with 95% confidence interval.

## Results

Ninety copies of the questionnaire pre and post intervention respectively were correctly and completely filled with a response rate of 100%. All respondents were female.

Table 1 shows the sociodemographic characteristics of the respondents. The average age was 46·5 years with a majority within the ages of 41–60 years (57·8%), married (72·2%) and with a secondary level of education (54·4%) being the highest level attained. The average length of time the respondents practiced as TBAs was 16 years with an average of ten birth deliveries per month. There was no statistically significant difference in the distribution between the sociodemographic characteristics of TBAs and the zones they reside and practice (*p* > ·05).

**Table 1:** Socio-demographic Characteristics of Traditional Birth Attendants by zones in Imo State

Variable	Category	Freq (%) ***N*** = 30			Freq (%) ***N*** = 90
		Orlu zone	Owerri zone	Okigwe zone	Imo State
Age group	20–40	9 (30·0)	8 (26·7)	12 (40·0)	29 (32·2)
*p* = ·516(df 2)	41–60	17 (56·7)	18 (60·0)	17 (56·7)	52 (57·8)
	Above 60	4 (13·3)	4 (13·3)	1 (3·3)	9 (10·0)
**Mean age (STD)**		**47·7 (11·8)**	**49·0 (11·6)**	**43·5 (12·9)**	46·**5 (**12·2**)**
Marital status	Single	1 (3·3)	1 (3·3)	4 (13·3)	6 (6·7)
*p* = ·349(df 2)	Divorced	2 (6·7)	0 (0)	0 (0)	2 (2·2)
	Widowed	8 (26·7)	7 (23·3)	2 (6·7)	17 (18·9)
	Married	19 (63·3)	22 (73·3)	24 (80.0)	65 (72·2)
Level of Education	Primary	10 (33·3)	4 (13·3)	5 (16·7)	19 (21·1)
*p* = ·192(df 4)	Secondary	16 (53·3)	18 (60·0)	15 (50·0)	49 (54·4)
	Tertiary	4 (13·3)	8 (26·7)	10 (33·3)	22 (24·4)
Years practiced as	< 1	2 (6·7)	1 (3·3)	2 (6·7)	5 (5·6)
TBA	1–9	9 (30·0)	7 (23·3)	14 (46·7)	30 (33·3)
*p* = ·249(df 4)	10–24	9 (30·0)	12 (40·0)	9 (30·0)	30 (33·3)
	Above 24	10 (33·3)	10 (33·3)	5 (16·7)	25 (27·8)
**Mean year (STD)**		17·**7 (**14·7**)**	18·**6 (**13·1**)**	12·**9 (**12·4**)**	16·**4 (**13·5**)**
No. of Deliveries	< 1	0 (0)	0 (0)	1 (3·3)	1 (1·1)
Per Month	1–9	15 (50·0)	18 (60·0)	14 (46·7)	47 (52·2)
*p* = ·669(df 2)	10–24	14 (46·7)	12 (40·0)	14 (46·7)	40 (44·4)
	Above 24	1 (3·3)	0 (0)	1 (3·3)	2 (2·2)
**Mean delivery (STD)**		**11·2 (6·5)**	**9·4 (6·0)**	**10·6 (6·9)**	10·**4 (**6·5**)**

**Fig. 1:**
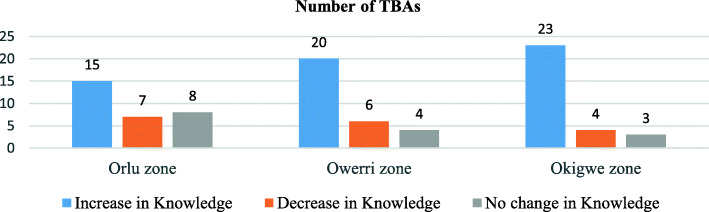
Number of TBAs by zones with “Change-No Change” in the knowledge levels Post Intervention

Table 2 shows the number of TBAs in each knowledge level category pre and post intervention. While in the pre intervention period, the majority of the TBAs had a moderate level of immunization knowledge (58·9%), in the post intervention, the majority of the TBAs had a good level of immunization knowledge (71·1%). In the pre intervention period, there was no association (*p* > ·05) between the level of knowledge of the TBAs and the zones they reside and practice but post intervention, there appeared to be an association (*p* = ·027). Okigwe zone had the highest number of TBAs with an increase in knowledge level (Fig. 1).

**Table 2:** Level of Knowledge of TBAs by zones in Imo State-Pre and Post Intervention

Period	Knowledge level	Freq (%) ***N*** = 30			***p*** value (df)	Imo State Freq (%) N = 90
		Orlu zone	Owerri zone	Okigwe zone		
Pre-intervention	Poor	4 (13·3)	1 (3·3)	2 (6·7)		7 (7·8)
	Moderate	16 (53·3)	18 (60·0)	19 (63·3)	·861 (2)	53 (58·9)
	Good	10 (33·3)	11 (36·7)	9 (30·0)		30 (33·3)
Post- Intervention	Poor	2 (6·7)	0 (0)	0 (0)		2 (2·2)
	Moderate	12 (40·0)	5 (16·7)	7 (23·3)	^a^·027 (2)	24 (26·7)
	Good	16 (53·3)	25 (83·3)	23 (76·7)		64 (71·1)

^a^significant

Table 3 shows the effect of the intervention using a paired sample t test analysis. There was a statistically significant increase in the TBA’s knowledge of immunization overall and across the zones after the workshop intervention (*p* < ·05).

**Table 3:** Effect of the intervention on knowledge of TBAs by zones in Imo State (Paired sample t test)

Area	Mean diff	Std Dev	95% CI	t	df	p
Orlu	1·9	4·2	− 3.429 -0.305	2·44	29	·021^a^
Owerri	2·5	3·9	−3.954 -1.046	3·52	29	·001^a^
Okigwe	3·1	3·2	−4.306 -1.894	5·26	29	·000^a^
Imo State	2·5	3·8	−3.281 -1.697	6·24	89	·000^a^

^a^significant

**Fig. 2:**
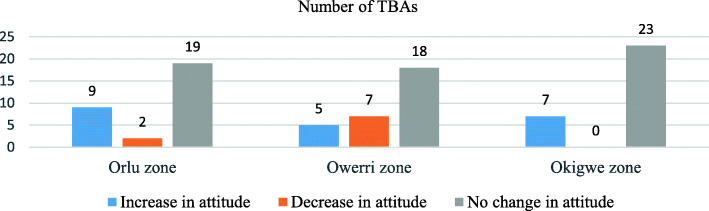
Number of TBAs by zones with “Change-No Change” in the attitude levels Post Intervention

Table 4 shows the number of TBAs in each level of attitude category pre and post intervention. Majority of TBAs in the pre (71·1%) and post (82·2%) intervention periods, had a good level attitude towards immunization. However, in both the pre and post intervention periods, there were no associations (*p* > ·05) between the level of attitude of the TBAs and the zones they reside and practice. Orlu zone had the highest number of TBAs with an increase in attitude levels while Okigwe zone had the highest number of TBAs with no change in attitude levels after the intervention (Fig. 2).

**Table 4:** Level of attitude of TBAs by zones in Imo State-Pre and Post Intervention

Period	Attitude level	Freq (%) N = 30			***p*** value (df)	Imo State Freq (%) N = 90
		Orlu zone	Owerri zone	Okigwe zone		
Pre-intervention	Poor	1 (3·3)	1 (3·3)	1 (3·3)		3 (3·3)
	Moderate	10 (33·3)	4 (13·3)	9 (30·0)	·187 (2)	23 (25·6)
	Good	19 (63·3)	25 (83·3)	20 (66·7)		64 (71·1)
Post- Intervention	Poor	0 (0)	1 (3·3)	0 (0)		1 (1·1)
	Moderate	4 (13·3)	6 (20·0)	5 (16·7)	·587 (2)	15 (16·7)
	Good	26 (86·7)	23 (76·7)	25 (83·3)		74 (82·2)

**Fig. 3:**
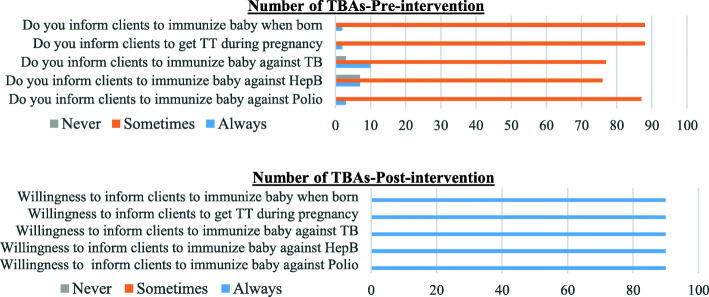
Frequency of practice and willingness to practice information exchange with clients on Immunization pre and post intervention respectively

Table 5 shows the effect of the intervention on the level of attitude using a paired sample t test analysis. After the intervention, there were statistically significant increases in positive attitude towards immunization among TBAs residing and practicing in Orlu and Okigwe zones. However, overall, the increase in positive attitude was not statistically significant (*p* = ·080). In Fig. 3, it was observed that, most TBAs in the pre-intervention period, only “sometimes” inform their clients about immunization but in the post intervention period all the TBAs were now willing to “always” inform their clients about immunization.

**Table 5:** Effect of the intervention on attitude of TBAs by zones in Imo State (Paired sample t test)

Area	Mean diff	Std Dev	95% CI	t	df	p
Orlu	0·5	1·3	−1.011 -0.056	2·28	29	·030^a^
Owerri	0·2	1·8	−0.461 0.861	0·62	29	·541
Okigwe	0·4	0·8	−0.738 -0.128	2·90	29	·007^a^
Imo State	0·3	1·4	−0.543 0.031	1·77	89	·080

^a^significant

## Discussion

This project was designed to assess the effect of a culturally adapted audio-visual workshop intervention to empower traditional birth attendants towards promoting the uptake of maternal and neonatal immunizations.

A number of studies [18–20] have shown that most TBAs are middle aged or older married women, with experience in child birth and they are highly respected in the rural communities they practice in. This was similarly observed in the present study where the TBAs were all women and the average age was 47 years with an average duration of 16 years practice as a TBA, performing an average of ten birth deliveries per month within their communities.

It was also observed that 72% of the TBAs were married. In the context of culture, especially within the rural communities among the women folk, married women are respected and furthermore, as TBAs, they have a long and consistent history of assisting birth deliveries in a friendly and caring manner that are readily available and affordable. This has endeared them to the community where they practice with wide spread social and cultural acceptance. This acceptance and intimate relationship they have established over the years make them uniquely positioned to act as effective agents to dispel the erroneous cultural and traditional beliefs and misconceptions about immunization which appear to be more evident in rural communities.

In spite of their unique position in the communities, the level of education of the TBAs is a challenge as studies have reported TBAs having no formal education or having some primary or some secondary education [18, 20]. In the present study, the majority of the TBAs had either a primary or a secondary education as the highest level attained but it was also observed that up to one quarter of the TBAs had a tertiary education. Similarly, a study in Nigeria [17] also reported having TBAs with a tertiary level of education. This observation may not be unconnected with the high unemployment rate in Nigeria for graduates of tertiary institutions; and probably, this has forced some of them to relocate to their communities and engage in traditional birthing activities as a means of livelihood. Notwithstanding the proportion of TBAs with a tertiary education, the majority still have either no or incomplete education experience, compounded by differing traditional and cultural beliefs. This poses a challenge in the development of effective communication materials that would impact significant knowledge on a maximum proportion of recipients with differing customs and traditions.

Nevertheless, the present study took into cognizance the level of literacy, language barriers, customs and traditions in the development of the audio-visual communication content but however, in the study’s post intervention period it was observed that there was a statistically significant association between the level of knowledge of the TBAs and the zones they reside and practice; as this, may have been influenced by the differing traditional and cultural beliefs. This further highlights the challenges of developing a uniform communication content for a mix of clusters characterized by low literacy and varying cultural and traditional beliefs.

Despite these challenges of the audio-visual workshop intervention, its effect on knowledge was statistically significant across the zones and in the total population of TBAs. This further emphasizes that, effective training of TBAs with appropriate materials can improve knowledge as similarly reported in other studies with training interventions, where knowledge had resulted in improved maternal and neonatal outcomes [12, 17, 18, 21].

The knowledge acquired by the TBAs may empower them to be at least willing to effect change as was observed in the present study where all the TBAs post intervention were now willing to encourage the uptake of immunization among their clients.

The study had limitations such as the content of the workshop communication messages. This was developed specifically for the traditional birth attendants by taking into cognizance their varying literacy levels and different cultural and traditional beliefs. However, communicating a unifying message with an appropriate mix of information and clear understanding for this diverse group of participants was still challenging. The resultant less than perfect uniform messaging could have impacted on the degree of knowledge acquisition, positive attitude development and invariably, their willingness to promote immunization. Another limitation was the translation of the questionnaire from English to the native language of Igbo by the interviewers when administering the questionnaire to participants not fluent in English language. This could have introduced interviewer bias in spite of the training conducted for the interviewers before the workshop. The challenge was the lack of equivalent words or concepts for some expressions and this could have impacted on the respondent’s interpretation and understanding and therefore, their knowledge and attitude towards immunization.

## Conclusion

It is imperative for traditional birth attendants in resource-limited settings to be knowledgeable about immunization and its benefits, and also be willing to encourage its uptake among their clients and their newborn.

This study highlights the empowerment of TBAs as a complimentary strategy for improving immunization especially in rural areas where they are predominantly involved with pregnant women and the birthing process. Also, it provides some insights for policy makers of the potential roles and impacts TBAs can specifically play in improving immunization uptake and reducing the prevalence of vaccine preventable diseases in our environment when empowered through appropriate training.

## Supplementary Information

**Additional file 1.**

## Data Availability

The data can be made available from the corresponding author under reasonable request.

## Abbreviations

TBA: Traditional Birth Attendants
MDG: Millennium Development Goals
TT: Tetanus Toxoid
TB: Tuberculosis
HepB: Hepatitis B
