# Supplementary material for: Empowering traditional birth attendants as agents of maternal and neonatal immunization uptake in Nigeria: a repeated measures design
Source: BMC Public Health. 2021 Feb 4;21:287. doi: 10.1186/s12889-021-10311-z (PMC7863363; doi:10.1186/s12889-021-10311-z)
Supplement: Supplementary file 1 — Additional file 1. [file 12889_2021_10311_MOESM1_ESM.docx]

**KAP STUDY FOR TBAs ON IMMUNIZATION NUMBER…………**

**PRE-WORKSHOP QUESTIONNAIRE I**

**SECTION A: SOCIODEMOGRAPHIC VARIABLES OF RESPONDENTS**

1. How old are you? : ______________
2. What is your Marital status?: **Single, Divorced/Separated, Widowed, Married**
3. What is your level of education?:  **Primary, Secondary, Tertiary**
4. How many years you practiced as TBA: ______________________
5. How many deliveries you do every week: __________________________

**SECTION B: KNOWLEDGE OF MATERNAL AND NEWBORN IMMUNISATION**

1. Does immunization of mother protect the baby from tetanus: **Yes No Don’t know**
2. Should mothers receive only 1 dose of tetanus toxoid to cover all pregnancies? **Yes No Don’t know**
3. Does immunization prevent mothers from getting pregnant? **Yes No Don’t know**
4. Should only healthy babies be immunized: **Yes No Don’t know**
5. Should Immunization be given to only sick newborns : **Yes No Don’t know**
6. Sick babies should not be immunized : **Yes No Don’t know**
7. Does immunization make newborn babies healthier: **Yes No Don’t know**
8. Does immunization of babies always have bad effects: **Yes No Don’t know**
9. Should you give immunization to a baby with fever? **Yes No Don’t know**

|  | VACCINE | TRUE | FALSE | I DON’T KNOW |
| --- | --- | --- | --- | --- |
| A | Oral Polio |  |  |  |
| B | Hepatitis B |  |  |  |
| C | Pentavalent |  |  |  |
| D | BCG |  |  |  |
| E | Measles |  |  |  |
| F | Yellow Fever |  |  |  |
| G | Pneumococcal conjugate |  |  |  |
| H | Vitamin A |  |  |  |

1. The following vaccines are usually given to newborn babies

**KAP STUDY FOR TBAs ON IMMUNIZATION NUMBER…………**

**PRE-WORKSHOP QUESTIONNAIRE II**

1. The following diseases can result if a child is not immunized.

|  | Disease | True | False | I DON’T KNOW |
| --- | --- | --- | --- | --- |
| A | Cholera |  |  |  |
| B | Tuberculosis |  |  |  |
| C | Measles |  |  |  |
| D | Staphylococcus infection |  |  |  |
| E | Pneumonia |  |  |  |
| F | Hepatitis |  |  |  |

**SECTION C: ATTITUDE TOWARDS MATERNAL AND NEONATAL IMMUNISATION**

1. Do you think immunization of mother and child is necessary?: **Yes No No opinion**
2. Do you think you should tell your clients about receiving immunization?: **Yes No No opinion**
3. Do you think immunization is stressful to the newborn baby and mother?: **Yes No No opinion**
4. Do you think cost should be a reason for your clients not to get immunization?: **Yes No No opinion**
5. Do you think distance should be a reason for your clients not to get immunization? **Yes No No opinion**
6. Do you think you should tell your fellow TBAs about encouraging immunization? **Yes No No opinion**

**SECTION D: PRACTICE OF INFORMATION EXCHANGE ON IMMUNISATION**

1. Do you inform your clients on immunization of their babies when born?: **Always/Sometimes / Never**
2. Do you inform your clients to get tetanus toxoid during pregnancy?: **Always / Sometimes / Never**
3. Do you inform your clients to immunize their Newborn babies from TB? **Always / Sometimes / Never**
4. Do you inform your clients to immunize their Newborn babies from Hepatitis B?:**Always/Sometimes/Never**
5. Do you inform your clients to immunize their Newborn babies from polio?: **Always / Sometimes / Never**

**KAP STUDY FOR TBAs ON IMMUNIZATION NUMBER…………**

**POST-WORKSHOP QUESTIONNAIRE I**

**SECTION B: KNOWLEDGE OF MATERNAL AND NEWBORN IMMUNISATION**

1. Does immunization of mother protect the baby from tetanus: **Yes No Don’t know**
2. Should mothers receive only 1 dose of tetanus toxoid to cover all pregnancies? **Yes No Don’t know**
3. Does immunization prevent mothers from getting pregnant? **Yes No Don’t know**
4. Should only healthy babies be immunized: **Yes No Don’t know**
5. Should Immunization be given to only sick newborns : **Yes No Don’t know**
6. Sick babies should not be immunized : **Yes No Don’t know**
7. Does immunization make newborn babies healthier: **Yes No Don’t know**
8. Does immunization of babies always have bad effects: **Yes No Don’t know**
9. Should you give immunization to a baby with fever? **Yes No Don’t know**
10. The following vaccines are usually given to newborn babies

|  | VACCINE | **TRUE** | **FALSE** | **I DON’T KNOW** |
| --- | --- | --- | --- | --- |
| A | Oral Polio |  |  |  |
| B | Hepatitis B |  |  |  |
| C | Pentavalent |  |  |  |
| D | BCG |  |  |  |
| E | Measles |  |  |  |
| F | Yellow Fever |  |  |  |
| G | Pneumococcal conjugate |  |  |  |
| H | Vitamin A |  |  |  |

1. The following diseases can result if a child is not immunized.

|  | Disease | **TRUE** | **FALSE** | **I DON’T KNOW** |
| --- | --- | --- | --- | --- |
| A | Cholera |  |  |  |
| B | Tuberculosis |  |  |  |
| C | Measles |  |  |  |
| D | Staphylococcus infection |  |  |  |
| E | Pneumonia |  |  |  |
| F | Hepatitis |  |  |  |

**KAP STUDY FOR TBAs ON IMMUNIZATION NUMBER…………**

**POST-WORKSHOP QUESTIONNAIRE II**

**SECTION C: ATTITUDE TOWARDS MATERNAL AND NEONATAL IMMUNISATION**

17. Do you think immunization of mother and child is necessary? **Yes No No opinion**

18. Do you think you should tell your clients about receiving immunization?: **Yes No No opinion**

19. Do you think immunization is stressful to the newborn baby and mother?: **Yes No No opinion**

20. Do you think cost should be a reason for your clients not to get immunization?: **Yes No No opinion**

1. Do you think distance should be a reason for your clients not to get immunization? **Yes No No opinion**
2. Do you think you should tell your fellow TBAs about encouraging immunization? **Yes No No opinion**

**SECTION D: WILLINGNESS TO PRACTICE INFORMATION EXCHANGE ON IMMUNISATION**

23 Are you willing to inform your clients to immunize their babies when born? **Always/Sometimes/Never**

24 Are you willing to inform your clients to get tetanus toxoid during pregnancy? **Always/Sometimes/Never**

25. Are you willing to inform your clients to give their newborns BCG vaccine? **Always/Sometimes/Never**

26. Are you willing to inform your clients to give their newborns Hepatitis B vaccine? **Always/Sometimes/Never**

27. Are you willing to inform your clients to give their newborns Oral polio vaccine? **Always/Sometimes/Never**
